# Maternal Substance Use, Child Maltreatment Risk, and Child Welfare Policy: An Equality-Based, Child-Centered Framework

**DOI:** 10.7759/cureus.109174

**Published:** 2026-05-19

**Authors:** Joy Ivaschenko, Gavin L Brunsvold

**Affiliations:** 1 Psychiatry, Christ Health Center, Birmingham, USA

**Keywords:** abandonment, child abuse, child rights, parental substance abuse, post-traumatic stress disorder (ptsd)

## Abstract

Substance use within caregiving environments is associated with increased risk of child maltreatment and adverse developmental outcomes. Child welfare systems often emphasize family preservation and reunification; however, in certain contexts, these goals can conflict with the need to prioritize child safety when risk factors are not adequately addressed. This narrative review and policy analysis examines the intersection of maternal substance use, child maltreatment, and reunification practices through an equality-based, gender-neutral accountability framework, synthesizing evidence from a range of clinical, epidemiological, and child welfare studies.

A structured narrative review and policy analysis were conducted using PubMed, JAMA, the American Psychological Association, and Google Scholar, along with governmental and epidemiological reports. Literature addressing caregiver substance use, child maltreatment, trauma exposure, foster care, reunification, and long-term developmental outcomes was screened using predefined inclusion and exclusion criteria and synthesized narratively by thematic category. Exposure to caregiver substance use and associated forms of maltreatment (verbal, emotional, psychological, physical, and abandonment) is strongly and consistently associated with increased risk of long-term mental health disorders, behavioral dysregulation, and chronic health conditions. Reunification without sustained behavioral change and verified safety is often associated with higher levels of re-traumatization in children. Child welfare policies may benefit from emphasizing gender-neutral, sustained behavioral change prior to reunification and from prioritizing child-reported safety and long-term stability in reunification decisions over permanent re-homing.

## Introduction and background

Maternal caregiver substance use is associated with increased risk of unstable caregiving, child maltreatment, neglect, and adverse developmental outcomes, with a high incidence of recurrence with reunification. Child welfare systems emphasize family preservation and reunification because they believe it is better for children in the long term. However, growing up in unstable and abusive environments increases negative long-term outcomes for children. Family preservation and reunification often conflict with the need to prioritize child safety when risk factors are not adequately addressed. An additional consideration is whether prevention of abuse could be strengthened by reconsidering default reunification practices and considering long-term placement in stable home environments.

Studies support that the sooner a child is placed in a stable environment, preferably within six months, the more likely they are to be able to recover without long-term mental health issues. This raises important questions for policymakers and providers regarding how child welfare systems should balance parental rights and family preservation goals with the obligation to prioritize child safety and the child’s right to grow up without trauma in cases involving persistent instability or substance abuse. This is particularly sensitive and important because doing so can challenge traditional interpretations of maternal rights. This paper does not suggest that maternal caregivers are uniquely responsible for the maltreatment of children. However, the emphasis on maternal substance use and related forms of abuse reflects the central caregiving role mothers often occupy and the potential influence this may have on reunification decisions and long-term child mental health outcomes. The review synthesizes evidence regarding abuse prevalence, developmental consequences, intergenerational trauma, and the limitations of current reunification practices. Policy recommendations emphasize prevention, accountability, trauma-informed care, and sustained behavioral change to better protect children and support long-term outcomes.

This study was conducted as a narrative review and policy analysis examining the relationship between caregiver substance use, child maltreatment, trauma exposure, reunification practices, and long-term developmental outcomes in children. Because of heterogeneity in study design, populations, and outcome measures, findings were synthesized narratively rather than through meta-analysis. Because the review included observational studies, governmental reports, and narrative literature, formal meta-analytic bias scoring was not feasible. However, priority was given to peer-reviewed studies, longitudinal methodology, systematic review design, or nationally representative datasets. Findings were interpreted with consideration of potential reporting bias, limitations of the retrospective design, and variability in definitions across studies.

A structured literature search was conducted between January 2026 and March 2026 using the databases PubMed, PsycINFO, and Google Scholar. Additional governmental and epidemiological reports were identified through the Centers for Disease Control and Prevention, U.S. Department of Health and Human Services, and the Substance Abuse and Mental Health Services Administration. Search terms included combinations of “child maltreatment,” “adverse childhood experiences,” “maternal substance use,” “parental substance abuse,” “foster care reunification,” “child welfare,” “emotional abuse,” “psychological abuse,” “child neglect,” “abandonment,” “attachment trauma,” “termination of parental rights,” “trauma-informed care.”

Studies were included if they examined child maltreatment, caregiver substance use, trauma exposure, foster care, reunification, or long-term developmental outcomes; included pediatric or adolescent populations; were peer-reviewed empirical studies, systematic reviews, governmental reports, or epidemiological analyses; and were published in English. Studies were excluded if they focused exclusively on adult outcomes or did not address maltreatment, caregiving instability, or reunification outcomes. Titles and abstracts were screened for relevance prior to full-text review. Data extracted from included studies included study design, population characteristics, trauma or maltreatment type, reunification outcomes, mental health outcomes, and major findings related to child safety or developmental effects. Evidence was synthesized narratively and grouped into the following thematic categories: substance use and child maltreatment; emotional and developmental consequences of abuse; reunification and foster care outcomes; attachment disruption and abandonment; and policy implications related to child safety and permanency.

## Review

Child maltreatment and substance abuse

Child maltreatment, including verbal, emotional, psychological, sexual, and physical abuse, abandonment, neglect, and exposure to substance use, is associated with significant long-term physical and psychological consequences [1]. When substance abuse occurs within a maternal caregiving role, the effects on children are often compounded by unstable caregiving patterns, neglect, and emotional, verbal, mental, or physical maltreatment, making child safety more difficult to ensure. These conditions raise concerns regarding how child welfare systems balance parental rights, reunification goals, different legal standards applied to fathers versus mothers, and child safety in cases involving persistent instability or substance use [2].

This study does not suggest that maternal caregivers are uniquely responsible for maltreatment. Evidence demonstrates that fathers and other caregivers also contribute significantly to child abuse and neglect [3]. However, the emphasis on maternal abandonment and substance use reflects the role mothers often play in primary caregiving, with the potential pitfalls of trauma and reunification decisions within many child welfare systems based on the maternal role over child safety. An equality-based framework for child rights and placement emphasizes that caregiving standards should be applied consistently across parents, regardless of gender, because children have the right to grow up without trauma. Women’s rights do not require the minimizing or excusing of harmful behavior and thus should encourage equal legal standards. Applying reduced legal standards to maternal neglect or abuse undermines both gender equality and child safety by reinforcing discriminatory assumptions that women require lesser expectations to maintain motherhood, thereby exposing children to continued harm under the guise of maternal rights [4]. Raising standards for maternal fitness supports the protection of children. Table 1 presents the themes presented in this study.

**Table 1:** Summary of major themes CDC: Centers for Disease Control and Prevention; HHS: Health and Human Services; SAMHSA: Substance Abuse and Mental Health Services Administration; PTSD: Post-traumatic stress disorder; ACE: Adverse childhood experiences.

Theme	Major findings	Representative sources
Caregiver substance use	Associated with increased neglect, instability, and foster care placement	CDC, HHS, SAMHSA reports
Emotional/verbal abuse	Linked to depression, anxiety, attachment disruption, and trauma symptoms	Child psychiatry and ACE studies
Reunification outcomes	Higher foster care reentry when substance use remains unresolved	Child welfare outcome studies
Attachment disruption	Associated with long-term relational instability and behavioral dysregulation	Attachment and trauma literature
Trauma exposure	Increased risk of PTSD, chronic disease, substance use, and suicidality	ACE and epidemiological studies

Emotional abuse and developmental trauma

High rates of emotional abuse among adolescents have been reported in national surveys, although prevalence estimates vary depending on definitions and methodology [5,6]. Emotional abuse is among the most common forms of maltreatment among girls and is associated with significant developmental consequences. These adverse experiences frequently result in long-term trauma. Despite existing legal and medical protections, gaps in identification and, more so, intervention persist, potentially allowing children to remain in unsafe environments. Although at-risk environments are increasingly identified, many interventions fail to meaningfully change children’s home conditions, allowing trauma to persist, as measures by the parent(s) are often implemented only temporarily for legal compliance rather than sustained change. This raises concerns regarding the effectiveness of current intervention approaches and long-term trauma outcomes for children.

Foster care and reunification outcomes

Alternative permanency options, such as adoption into stable environments or termination of parental rights for the unstable parent figure(s), warrant serious consideration and show increasing promise in improving long-term outcomes for children [6]. Exposure to adverse childhood experiences (ACEs) increases the likelihood of developing violent behaviors, substance use, risky sexual behaviors, mental health disorders, and suicidal ideation [7]. Although the full scope cannot be precisely quantified, research shows that children returned to environments associated with trauma increases the likelihood of continued trauma and consistently shows that abuse patterns are transmitted intergenerationally, particularly among girls, who may normalize experiences of violence and neglect into adulthood [5]. Thus, prevention, including primary interventions focused on preventing ACEs, remains the most effective strategy for reducing long-term maladaptive outcomes.

Current child welfare and family court frameworks emphasize reunification as the primary permanency goal 55% of the time, even in cases involving documented substance use and emotional, verbal, physical, or abandonment-related abuse [7,8]. Despite the “best interests of the child” standard, children’s legal rights remain limited. Reunification is often prioritized within the maternal rights framework despite ongoing safety concerns, including after substance use, abandonment, or repeated abuse, while men in similar circumstances are often held to higher standards and face less leniency when trying to gain back rights to their children. Children, particularly those younger than 14 years of age, are mandated to participate in therapy and supervised, or unsupervised, visitation despite distress or re-traumatization, even when reunification has not resulted in sustained behavioral change [9,10]. These findings raise concerns regarding whether reunification without sustained behavioral change (e.g., 12 months or more) adequately protects long-term child safety and emotional stability. Custody reinstatement based on procedural compliance rather than demonstrated emotional safety and long-term behavioral stability places children in abusive and emotionally suppressive environments [9,10].

Contrary to assumptions of therapeutic benefit, reunification without verified safety and sustained behavioral change contributes to increased depression, anxiety, attachment disturbance, emotional dysregulation, other somatic complaints, withdrawal, regression, and other trauma-related responses in children [7,11,12]. This list is not exhaustive, but it is disturbing and calls for reform within the medicolegal realms. Emotional and verbal abuse are associated with long-term disruptions in identity formation and self-worth. These behaviors may at times be misinterpreted as oppositional conduct rather than manifestations of trauma. It is reported that two-thirds of children, from a 17,000 participant study, experience major trauma as a child [13]. Approximately two-thirds of those children were diagnosed with a behavior or conduct disorder before a trauma [13]. This misinterpretation can contribute to additional stress and further psychological harm for the child.

Studies additionally show that children placed in foster care as part of the reunification process face a higher likelihood of reentry when parental mental health or substance use issues remain unresolved [14,15]. It is estimated that approximately two-thirds of all children who are placed in foster care or other family homing have at least one parent involved in substance use [16]. According to the U.S. Department of Health and Human Services, 74.8% of child protective service cases where children are removed involve neglect [17]. They also found that approximately 1,750 child fatalities were from abuse and neglect and that three-fourths of these cases involved at least one biological parent [17].

In households affected by substance use, disruption of supervision, boundaries, and caregiver stability increases the likelihood of negative developmental trajectories. Children of substance-involved parents demonstrate higher prevalence of anxiety, depression, attention, and conduct disorders, post-traumatic stress disorder, academic disengagement, insecure and unhealthy attachment, low self-worth, substance use disorders, relational instability, socioeconomic disadvantage, chronic physical illness, and later involvement with the justice system, all of which may have lasting consequences for socioeconomic outcomes [4]. These outcomes contribute to increased healthcare costs and perpetuate intergenerational poverty, homelessness, and malnutrition [18].

Abandonment and attachment disruption

Maternal abandonment is associated with significant developmental trauma in children, including chronic anxiety, depression, attachment disorders, and pervasive self-blame [19,20]. Research suggests abandonment activates the hypothalamic-pituitary-adrenal (HPA) axis, contributing to chronic stress responses, memory impairment, and impulse control difficulties that may persist into adulthood [21]. Abandonment by a parent is often preceded by other forms of abuse, including emotional abuse, neglect, and withdrawal of caregiving presence and support. Abandonment also contributes to long-term relational dysfunction, including difficulty forming secure attachments, relationship sabotage, rejection sensitivity, fear of intimacy, and maladaptive coping strategies. Individuals are at a high risk of developing personality disorders such as borderline personality disorder or dependent personality disorder, or engaging in behaviors such as substance use, self-harm, or eating disorders [18]. Repeated abandonment and reunification cycles are associated with exacerbated disorganized attachment, relational instability, maladaptive coping behaviors, and increased risk of later major mental health disorders [18,19]. Children subjected to repeated reunification cycles may be at a particularly elevated risk of sexual victimization due to impaired boundary formation and relational instability.

Grooming and sexual abuse risk

Grooming behaviors are a key risk factor for sexual abuse [9]. Grooming involves abusers desensitizing children to sexual content and physical contact while often evading detection. Effective prevention strategies must focus on identifying early signs of grooming behaviors, taking the signs seriously, and intervening promptly. Grooming tactics include favoritism, boundary violations, threats, exposure to sexualized content, physically grooming a child before it is age-appropriate, and normalization of sexual behavior [12]. Early intervention is critical to preventing escalation of grooming behaviors, which often remain unrecognized or unaddressed until severe psychological harm has already occurred [10]. Physical and sexual abuse may be minimized due to the lack of visible evidence despite profound neurodevelopmental impact [11]. These forms of maltreatment are associated with long-term alterations in brain and nervous system development, with severe consequences persisting into adulthood. Abuse exposure is also associated with accelerated biological aging, autoimmune conditions, and other chronic multisystem health disorders [22]. Psychological abuse, which often accompanies sexual abuse, contributes to hypervigilance, anxiety, and maladaptive coping patterns. Abuse, particularly maternal abuse, in all its forms consistently leads to negative outcomes for the children involved. So, what are the best medicolegal practices to help reduce the harm many children experience daily in our society?

Policy recommendations - equality, accountability, and child protection

To align child welfare systems with principles of equity and harm prevention, policies should emphasize evidence-based, gender-neutral standards that prioritize child safety, their individual rights, and long-term well-being. These recommendations are informed by literature on trauma, substance use, and child maltreatment [3,18,23].

Gender-Neutral Parental Fitness Standards

Parental fitness assessments should be applied equally across caregivers, regardless of gender. Evaluations should prioritize demonstrated behavior, sustained recovery, and the child’s emotional and physical safety rather than assumptions related to parental roles [1,2]. Evidence supports that 53.7% of abuse is from the female caregiver and thus supports a more neutral gender stance that considers the safety of the child over the gender of the caregiver/parent [17].

Recognition of Non-physical Forms of Maltreatment

Verbal, emotional, psychological abuse, neglect, and abandonment should be consistently recognized as actionable forms of maltreatment and should entertain the possible benefit of termination of parental rights to the child. These forms of harm are associated with significant developmental and mental health consequences and should be systematically evaluated in custody and reunification decisions [3,11].

Evidence of Sustained Behavioral Change

Reunification decisions should be based on sustained, verifiable behavioral change rather than procedural compliance alone. Indicators may include documented sobriety through objective laboratory testing, stable living conditions, and consistent, non-abusive caregiving behaviors for at least 12 months [14,23]. Eyewitnesses, recorded statements, and texts should also be evaluated in the evidence-gathering phase to support claims of behavioral changes, as people will often self-incriminate.

Inclusion of Child Perspective

Children should be provided with developmentally appropriate opportunities to express preferences regarding contact and reunification through extended evaluations conducted by trained professionals. Their reported experiences and sense of safety should be meaningfully considered in decision-making [14,24].

Limitations of Forced Contact

Court-mandated visitation or therapy should be carefully evaluated when there is evidence of psychological harm or re-traumatization. Interventions should be individualized and guided by the child’s best interests rather than default reunification goals [10,11]. If regression occurs, then forced contact should be reduced or eliminated depending on the circumstances of the case, e.g., one parent willingly left with no intention of returning and then decided they wanted to be a part of the child's life again.

Trauma-Informed Training

Judicial and child welfare professionals should receive standardized training in trauma-informed care to improve recognition of trauma responses and reduce misinterpretation of child behavior [2,11]. This training should be interactive and involve testimonies of children who were not protected from preventable trauma so that the weight of the reality of outcomes can be fully felt by those making decisions and providing care.

Prioritization of Safety and Permanency

In high-risk cases involving persistent substance use or maltreatment, child safety and permanency should take precedence over reunification timelines. Prolonged instability is associated with adverse developmental and health outcomes [3,9]. Thus, a set time limit should be established so that children can be placed in a permanent home within no more than six months, to improve long-term mental health and overall wellness outcomes.

Accountability-Focused Advocacy

Policy and advocacy efforts should emphasize accountability and recovery support while maintaining consistent caregiving standards. Integrating child protection with equitable expectations across both genders of caregivers may improve both safety and long-term outcomes [22,25].

Limitations

This study has several limitations. First, this review was conducted as a narrative synthesis rather than a formal systematic review or meta-analysis, limiting the ability to quantify pooled effect sizes across studies. Second, the included literature varied substantially in methodology, definitions of maltreatment, follow-up duration, and outcome measurement. Third, many studies examining child maltreatment and reunification rely on observational or retrospective designs, which limit causal inference. Additionally, child welfare policy varies across jurisdictions, potentially limiting generalizability. Finally, publication bias and underreporting of abuse may influence the available literature.

Healthcare providers, particularly those in pediatrics and psychiatry, should be well-versed in recognizing the signs of child maltreatment, substance abuse, grooming, and behavioral risk factors. Early, coordinated intervention may significantly reduce the risk of trauma escalation and improve long-term outcomes for affected children.

## Conclusions

Prioritizing child safety in cases involving maltreatment and substance abuse is consistent with principles of equality, accountability, and child-centered welfare policy, and not gender preferences. It is pro-equality, pro-accountability, and pro-child. An evidence-informed, trauma-sensitive, and child-centered approach to child welfare policy is essential for safeguarding children while also maintaining equitable standards for caregivers. By emphasizing accountability and behavioral change, we can better protect children from harm and support both their physical and psychological development. A feminist framework aligned with child abuse and neglect prevention may benefit from reconsidering frameworks that unintentionally minimize certain forms of maltreatment that excuse abuse under maternal rights and instead promote equal parental standards, prevention-focused intervention, verified safety prior to reunification, higher consideration of termination of parental rights in abuse situations, and child-centered legal decision-making.
